# Mental health literacy, mental health experiences and help-seeking behaviours of Chinese elite athletes: a qualitative study

**DOI:** 10.3389/fpubh.2024.1391597

**Published:** 2024-05-15

**Authors:** Danran Bu, Chun-Qing Zhang, Jing-Dong Liu, Zhe Han, Xiang Wang, Zhijian Huang

**Affiliations:** ^1^School of Physical Education, Hubei University, Wuhan, China; ^2^Department of Psychology, Sun Yat-sen University, Guangzhou, China; ^3^Department of Physical Education, Sun Yat-sen University, Guangzhou, China; ^4^Key Laboratory of General Administration of Sport of China, Hubei Institute of Sport Science, Wuhan, China; ^5^The Department of Sport, Physical Education and Health, Faculty of Social Science, Hong Kong Baptist University, Kowloon Tong, Hong Kong SAR, China

**Keywords:** mental health literacy, mental health experiences, help-seeking, Chinese elite athletes, a qualitative study

## Abstract

**Introduction:**

Elite athletes' help-seeking on mental health might be influenced by their mental health literacy (MHL) and mental health experiences. The current study aimed to explore the MHL, experiences and help-seeking behaviours among elite athletes using a qualitative approach.

**Methods:**

Face-to-face semi-structured interviews were conducted among 20 Chinese elite athletes, 12 coaches, and 5 team officials. Interview data was analyzed using content analysis.

**Results:**

Seven main themes emerged from the analysis. The current study revealed that Chinese elite athletes suffered from various mental health issues and athletes' MHL levels, help-seeking attitudes and intentions, Chinese sports environments, and Chinese cultural background could impact their help-seeking behaviours.

**Conclusion:**

Support for Chinese elite athletes' mental health and help-seeking requires improvement.

## Introduction

The importance of elite athletes' mental health has been well-recognised by several originations, such as the International Olympic Committee (IOC) (1), the International Society of Sport Psychology (ISSP) (2), the European Federation of Sport Psychology (FEPSAC) (3), the American Medical Society for Sports Medicine (AASQP1) (4), and researchers (5, 6). Yet, mental health issues among elite athletes remain prevalent, which may have negative impacts on athletes' mental wellbeing, performance, and development (5, 7–9). Elite athletes often struggle with mental health issues such as anxiety disorders, depression, eating disorders, sleeping issues and other mental health issues (1, 10–12). Specifically, the prevalence of anxiety disorder in elite athletes ranges from 6.0% (clinically confirmed) (13) to 14.6% (self-report) (14) and depressive symptoms from 4% (13) to 68% (15). The estimated prevalence of eating disorders and/or eating disorders in athletes is between 0 and 19% in men and 6 and 45% in women (16). More than half of U.S. college athletes regularly experience sleep deprivation, especially during the season, with 50% sleeping <7 h a night and 79% sleeping <8 h (17). An investigation of 210 Chinese elite athletes revealed that over 30% of these athletes suffered from depression and anxiety (18). Additionally, compared with team sports athletes, athletes in individual sports tend to suffer from higher levels of depression and anxiety (19, 20).

Despite that elite athletes may have mental health issues, they often delay seeking professional help for years (21, 22). Help-seeking, as used in the context of mental health, is an adaptive coping strategy that entails looking for outside support to address mental health issues (23). It is a multifactorial process that involves how cognition (e.g., beliefs, attitudes) influences intentions and subsequent behaviours and focuses on communication and action for advice, treatment and support for a health problem (23). Previous research has found that 86% of Chinese elite athletes have never sought professional help for mental health issues (24). Athletes may encounter certain obstacles in the elite sport environment that affect their attitudes and, ultimately, make them less likely to ask for assistance (25). How to encourage athletes' help-seeking behaviours has become a high priority for research and for policy or programme initiatives (23).

Some studies have shown that poor mental health literacy (MHL) is a major barrier to help-seeking behaviours in athletes (26, 27). MHL is defined as the knowledge and beliefs about mental disorders that can assist individuals in recognising, managing or preventing mental issues (28). It is common that elite athletes often lack knowledge of the symptoms of mental disorders, such as the difference between fatigue caused by physical exhaustion and fatigue caused by depression or anxiety (21). In particular, 70% of Chinese elite athletes showed low levels of MHL (24). Therefore, prompt action to improve their MHL is needed (25).

Stigmatising attitudes towards mental health influence athletes' help-seeking behaviours when suffering mental health problems. It refers to the negative attitudes towards individuals or groups who exhibit traits or behave in ways that are perceived as inappropriate or insufficient by the broader community (29, 30), which further divided into can be public stigmas and self-stigmas. public stigma is the notion expressed by the general public that the stigmatised group is socially unacceptable (31). Self-stigma is the devaluation of an individual's self-worth and self-efficacy that happens when they encounter mental health problems as a result of stigma from the public (31). Sports contexts sometimes discourage showing weakness, hence there is a significant level of stigma associated with mental health concerns in sports culture (32–34).

The avoidance of help-seeking when suffering from mental health issues can be explained by the psychological theory of behaviour change (35). Specifically, the help-seeking theory (36) and the theory of planned behaviour (37) provided an effective theoretical underpinning for this study. According to the help-seeking theory, the help-seeking theory comprises four steps: (1) problem recognition and assessment; (2) symptom expression and support need; (3) assistance sources' accessibility; and (4) readiness to look for and tell sources (36). The first and the second steps are associated with MHL and help-seeking attitudes (38). Additionally, according to the TPB, attitudes predict intentions, which in turn predict behaviours (37). In summary, the above two theories include a person's knowledge and perceptions of available sources of help and his or her attitudes, intentions and behaviours about help-seeking (36).

Chinese cultural background and sports environment is different from Western countries. Chinese elite athletes who are trained in China's “Whole Nation” system may experience various unique mental health issues and risk factors due to the highly competitive collective training environment (32). In particular, their training and competition experiences are deeply associated with the significance of their national identity and culture. Indeed, the cultural differences between Western and Eastern countries are relatively large, especially in mental health. Take the example of the US and China: in the US, the purpose of mental health reflects individualism, namely, “assist individuals to overcome obstacles to their personal growth, wherever these might be encountered, and … [achieve] optimum development of their personal resources” (39). This objective is mainly focused on individuals and their personal rights and interests (40). In contrast, the objectives of mental health services in China are “to enhance public mental health, facilitate social stability as well as interpersonal harmony, and improve public well-being … and to foster personal morality, promote coordinated … economic and social development, nurture and exercise socialist values and principles” (41). This definition has three aspects: emphasising public health, facilitating harmony, which involves individuals and society, and emphasising morality and social responsibility (40). It should be noted that the different perspectives on mental health are highlighted in different cultural values, especially in terms of individualism and collectivism (40). However, most studies focusing on mental health issues, MHL and help-seeking behaviours are from developed countries (42, 43). Research in developing countries, e.g., China, which may have a different culture, may further enrich the literature (42).

Furthermore, previous relevant qualitative studies are often based on a single perspective, such as coaches or athletes. For instance, Wood et al. (27) focused on the experiences of mental health issues and help-seeking from the perspective of male professional football athletes. However, such analysis from one perspective may not capture the full image of the story (44, 45). Thus, this paper aims to understand the mental health experiences, MHL and health-seeking behaviours of Chinese elite athletes from the triangulated perspectives of elite athletes, coaches, and sport team officials.

## Methods

### Philosophical orientation

Researchers should acknowledge their epistemological and philosophical positions when performing qualitative data analyses (46). In this study, the principles of critical realism was followed (47), which is based on a realist ontology and a modified dualist/objectivist epistemology. Critical realism assumes that subjects' understanding of truth cannot be objectively or fully measured, and it emphasises that the influence of the researcher should be controlled to produce knowledge as objectively as possible (48). Therefore, critical realism allowed us to acquire insights into the experiences, knowledge of mental health and help-seeking behaviours of the participants, and to do so with consideration of various perspectives.

### Participants

Elite athletes, coaches, and team officials from different sport training centres in two Chinese provinces were invited. Interviewees were selected using the following criteria: (a) elite athletes competing at regional, national, or international levels; (b) coaches competing at regional, national, or international levels; and (c) team officials in administrative roles who served as heads of sport training centres. No incentives were given for participation and they can voluntarily decide whether or not to participate. Conditional on the acceptance of invitation, interview appointments were made. As athletes in individual sports have been shown to experience a higher incidence of mental health symptoms than athletes in team sports (19, 20, 49), this study involved elite athletes in individual sports only. All the participants were recruited from Guangdong and Hubei Provinces in Mainland China.

### Procedure

Convenience sampling method was used in this study. The sampling process stopped when the number of interviewed participants reached the saturation of qualitative research (50). Signed letters of consent were received from the participants before the interview. Additionally, the parents of participants under the age of 18 provided their informed consent. All of the participants were promised that their data would be kept confidential. No participants refused to participate or dropped out of the study after the commencement of the interview, although they were allowed to drop out of the interview if any topic they felt uncomfortable or unwilling to discuss.

The interview guidelines included open-ended questions regarding the experience of mental health issues caused by training and competition, knowledge of mental health issues and risk factors, knowledge of help available, elite athletes' help-seeking attitudes, intentions and behaviours, and related factors that may affect their behaviours. Therefore, the interview questions involved (1) what do you think mental health is? (2) have you/athletes had mental health experience? (3) what are the help-seeking behaviours among athletes? (4) what are the athletes' knowledge of mental health issues and risk factors? (5) what are the help-seeking attitudes and intentions among athletes? (6) are there any other factors that impact help-seeking behaviours? They were encouraged to be as open and honest as possible concerning their past experiences. Because participants were not informed about the operationalised definition of mental health issues, they may hold different understanding of mental health issues.

Face-to-face semi-structured interviews were conducted to explore elite athletes' experiences of mental health issues, MHL, potential factors related to help-seeking behaviours. The interviews with the athletes and coaches were conducted in the counselling rooms after training sessions. The interviews with the team officials were conducted in their offices. The interviews were audio-taped, and all of the audio recordings were later transcribed verbatim. The durations of the interviews varied from 21.42 to 60.2 min. A code was used for each participant, and the participants' names were not mentioned in the results or the reports. Ethical approval was received from the Research Ethics Committee of XXX University [masked for review].

### Data analysis

The data was analysed with the assistance of NVivo Qualitative Data Analysis Software, Version 11 (QSR International Pty Ltd 2015). The content analysis approach was followed in line with the analytic induction method recommended by Elo and Kyngäs (51). Qualitative content analysis is a research method that involves the systematic categorisation process of coding and identification of themes or patterns to enable the subjective interpretation of text data content (52). It focuses on the features of language as a means of communication, paying particular emphasis to the text's content or context (53–56). Text data can come from interviews, focus groups, narrative answers, open-ended survey questions, print media like books, journals, or manuals, or it can come from verbal, print, or electronic sources (57). Specifically, there are three steps: creating tags, creating categories and analysing categories. First step, meaning units in the form of tags were created representing useful information from the participants. This procedure involved dividing the interview texts into meaning units and tagging them with temporary names that represented the key concepts of the text segments. Second step, categories were created by combining similar meanings and to determine labels that would describe the broader topics for each related cluster of tags. Tags may belong to more than one category. Third, the step of analysing categories involved identifying the similarities and differences in content between categories (58, 59). The whole coding process was conducted by two independent coders, separately. Discrepancies were resolved via discussion between the two coders to reach an agreement (27). Yardley's four guidelines: sensitivity to context, commitment and rigour, transparency and coherence, and impact and importance (60, 61) were also followed to improve the trustworthiness.

## Results

### Demographic information

In total, 20 athletes (*M* age = 22.5, *SD* = 4.37, range = 15–32; male = 10), 12 coaches (*M* age = 39.08; *SD* = 10.56, range = 26–59; male = 8) and five team officials (*M* age = 47; SD = 4.47, range = 42–54 years; all males) took part in the interviews. The recruited athletes were from shooting (three females and four males), badminton (two females), table tennis (two females and two males), boxing (one male), weightlifting (one female and one male), swimming (one male), gymnastics (two males), and long jump (one male), with training experience ranged from 4 to 15 years (*M* = 10.32; *SD* = 3.09). The elite athletes either competed at the national (*n* = 11) or international levels (*n* = 9). The recruited coaches were from shooting (one female and two males), badminton (two males), gymnastics (one male), jump (two males), martial (one male), diving (one male), and fencing (two males). The coaches had between 3 and 30 years (*M* = 13.17; *SD* = 9.73) of coaching experience, and competed at the regional (*n* = 5), national (*n* = 2) or international levels (*n* = 5). The team officials were the heads of the sports programmes for the participating athletes and coaches, namely badminton, table tennis, martial arts, track and field, and water sports programmes.

### Themes

Seven main themes involving thirteen sub-themes emerged from the content analysis. Seven themes were (1) mental health experiences; (2) mental health literacy; (3) help-seeking attitudes; (4) help-seeking intentions; (5) help-seeking behaviours; (6) Chinese sports environment and (7) Chinese cultural background. The results in terms of themes, sub-themes and condensed meaning units are presented in Table 1.

**Table 1:** The themes, sub-themes, and condensed meaning units (MU) of mental health experiences, mental health literacy, and help-seeking behaviours of Chinese elite athletes.

**Themes**	**Sub-themes**	**Condensed MU**
Experiences of mental health	Experiences of mental health issues	MU 1: depressed mood
		MU 2: competitive anxiety
		MU 3: sleep-related concerns
		MU 4: weight control issues
	Impact on performance	MU 1: performance
Mental health literacy	Knowledge of mental health information	MU 1: inadequate knowledge of mental health
		MU 2: limited approaches to acquire mental health knowledge
		MU 3: some approaches to acquire mental health knowledge
	Knowledge of help available	MU 1: limited knowledge of help available
		MU 2: knowledge of help available
Help-seeking attitudes	Stigma	MU 1: public stigma
		MU 2: self-stigma
	Positive attitudes	MU 1: positive attitudes
Help-seeking intentions	Help-seeking intentions from others	MU 1: seeking help through formal approaches
		MU 2: seeking help through informal approaches
	Self-help intention	MU 1: self-help intention
Help-seeking behaviours	Seeking help from others	MU 1: seeking help through formal approaches
		MU 2: seeking help through informal approaches
	Self-help strategies	MU 1: positive self-help strategies
		MU 2: negative self-help strategies
Chinese sports environment	Social support	MU 1: SUPPORT from coaches, teammates, friends, parents, leaders and sports psychologists
	Chinese sports system	MU 1: good relationships with others
		MU 2: bad relationships with others
		MU 3: positive training environment
		MU 4: negative training environment
Chinese cultural background	Chinese cultural background	MU 1: positive influence of Chinese culture
		MU 2: negative influence of Chinese culture

MU, meaning units.

### Experiences of mental health

Regarding the experience of mental health, the participants in all three groups admitted that the majority of elite athletes suffer from various mental health issues, and these mental health issues might lead to poor performance.

#### Experiences of mental health issues

The participants across three groups perceived depressed mood and anxiety about performance to be the most prevalent mental health concerns among elite athletes. Sleep-related concerns were emphasised by the athletes and coaches. Weight control issues were also highlighted by the athletes and team officials.

##### Depressed mood

Many elite athletes reported having experiences of low mood, tearful situation, and feelings of worthlessness during training. The coaches and team officials highlighted that these kinds of emotions are associated with injuries, weight control issues and dissatisfaction over past performance.

I feel like I'm having a breakdown. I can't even train on the training ground. When I start training, I can't stop myself from crying. I even cried in a dream. I feel depressed, and am suffering as a result of training. I can't stand it. And I feel worthlessness about my training, about this game (Athlete 14).

One athlete got injured. She was afraid to practise the diving technique related to her injury. When I asked her to do this diving technique, she cried. This situation lasted for several months (Coach10).

Athletes suffer from depressive symptoms for various reasons, such as injury or weight control, but the main reason is poor performance (Team official 1).

##### Competitive anxiety

In particular, anxiety often rise in elite athletes before competitions, especially for significant major events. Such pre-competitive anxiety, as pointed out by coaches and team officials, can cause muscle tension, fatigue, loss of appetite and sleep problems, which may reduce athletes' performance.

When I'm stressed, I tend to worry about training. And I can't sleep, I don't sleep all night, and I feel anxious. But when I can't sleep, I get more and more anxious. I don't know what to do (Athlete 14).

Some athletes might have pre-competitive anxiety. They can not perform well as usual due to muscle tension and sleep difficulties (Coach 9).

They felt anxiety before the competitions, especially for those significant upcoming competitions, such as the Whole National Game and championship which recognised as the most important competitions each year. Muscle tension, loss of appetite and some other physical symptoms could be observed (Team official 4).

##### Sleep-related concerns

The elite athletes and coaches recognised that insufficient sleep is likely to happen during the nights before competitions. Some athletes had been suffering from sleeping issues for a long time, and experienced high levels of fatigue. These issues were believed to affect the athletes' participation and performance in their sports.

I just can't sleep. It's been a long time, about a year. I always feel tired. If you can't sleep well, you can't recover. Thus, I can't train well during the day (Athlete 10).

If athletes do not sleep well for a long time, it will affect their training qualities and competitive ability. Because they will feel tired all day and will not focus on training (Coach 3).

##### Weight control issues

The elite athletes and team officials identified that athletes might develop eating and weight-control issues that could affect their physical functions. Such issues may have psychological consequences, especially for those engaged in weigh-in sports. However, eating and weight control difficulties were not emphasised by coaches.

It is believed that weight control could influence the competition and mental health issues. It is hard to prepare for the competition after you lose your weight because you do not have enough energy to prepare. Negative emotions emerged. It could be an important issue for us (Athlete 3).

In elite training, athletes suffer from eating and weight control problems. For example, athletes in weigh-ins sport, such as boxing, wrestling and lightweight rowing, need to control their weight for competitions. However, they may suffer from anxiety due to their strict requirements and those of their coaches, thus inadequate intake and negative psychological states due to weight control can affect their performance (Team official 5).

#### Impact on performance

##### Performance

The participants felt that mental health issues might result in poor performance in training, which could hinder athletes' performance in competitions. The depressed mood, competitive anxiety, sleep-related concerns, and weight control issues negatively impact on performance, especially in the major international or national sports events.

All of us are worried about poor performance, especially in the major international or national sports events, such as the Whole National Games. Firstly, we were afraid of being disappointed by families and coaches. Secondly, the benefits, including money, fame, and, even your future, would push you to think about the competition, the results. The more pre-competition anxiety had, the poor performance you got (Athlete 2).

Athletes worry about a competition if they can't turn their stress into motivation immediately. With this negative thinking, their perception will worsen, which will increase their fear and worry. Thus, it can lead to poor performance and low participation in training (Coach 3).

I think the competition anxiety and stress-related issues could lead to poor performance (Team official 3).

### Mental health literacy

Although concerns about mental health issues have increased within sporting community, the participants admitted that their knowledge of mental health issues, the approaches they used to acquire this knowledge and their knowledge of the available helps were not sufficient.

#### Knowledge of mental health issues

The participants across groups admitted that athletes do not have adequate knowledge of mental health issues, mental health risk factors, and access to possible sources. Some admitted that their possible sources of knowledge are coaches, sports psychologists, parents, friends, teammates, the Internet, and books, which enabled them to develop knowledge to be aware of mental health issues. Some of them perceived the need for education on mental health information. Additionally, coaches and team officials emphasised the education level of elite athletes might have impacted their awareness of mental health. Team officials further suggested that enhanced interest in mental health might be an appropriate means to promote awareness of mental health. Thus, education with the relevant knowledge needed to be conducted to enhance mental health in elite sport.

Because we haven't reached a very high level [of education], we probably don't know much about these things. Just like if I didn't know you, I would not know what sport psychology is. This means that understanding of mental health knowledge is limited in sport teams (Athlete 10).

Nowhere to get mental health knowledge (Athlete 11).

The athletes didn't have enough mental health knowledge. The awareness of mental health issues was highly relevant to the accumulation of knowledge, thus, they might not aware of the importance of mental health (Coach 1).

Limited approaches to getting relevant mental health knowledge (Coach 2).

The way to acquire knowledge in sports field was limited. How to enhance interest in sport psychology and get involve in leaning mental health was of importance (Team official 1).

#### Knowledge of available helps

In general, the participants agreed that education concerning the relevant resources for help is limited. The possible sources of help are coaches, sports psychologists, the Internet, and books. They revealed that Internet and books could facilitate access to information and services, but had concerns about their quality. Moreover, the emphasised relevant education should be offered to enhance elite athletes' knowledge of help available when suffering mental health issues.

I would like to learn from sport psychologist and psychological consulting. The information which is obtained from the website cannot effectively address my mental health issues, as it lacks individualized prescriptions or scientific treatment strategies (Athlete 10)?

There is an ineffective approach to acquire knowledge regarding mental health. The athlete might learn from the Internet, or coaches. But I am not sure it is enough for them (Coach 6).

Education related to these and counselling by sport psychologists may be the available approaches to seek help. In addition, they may gain relevant knowledge from the Internet, books, coaches and parents (Team official 5).

### Help-seeking attitudes

The majority of the interviewees agreed that negative attitudes exist towards elite athletes who seek help for mental health issues, and that both public stigmas and self-stigmas are involved. However, some of the participating athletes and coaches expressed positive attitudes towards elite athletes who seek help. These participants thought that everyone is at risk of experiencing mental health issues.

#### Stigmas

Stigmatising attitudes towards mental health was highlighted in all three groups. Both public- and self-stigmas were perceived. Specifically, the interviewees in all three groups felt that members of the public are likely to view athletes who seek help for mental health issues as weak and inadequate. This response indicated that the participants perceived a high level of public stigma regarding help-seeking. Furthermore, the athletes themselves said that they would feel ashamed if they had a mental health problem or if the public knew that they had sought help for a mental health problem.

Athletes who have mental health issues are thought to have less self-confidence. Athletes themselves are ashamed of having mental health issues, as the image they present to the public is supposed to be tough and strong (Athlete10).

I thought about why I didn't go to see a sport psychologist. The answer was that I was shy and embarrassed. I was afraid of being found out by my coach or teammates. I should be confident; I should be mentally strong (Athlete 5).

As far as I know, some parents worry about their children and do not recognise their mental health problems. They believe that their children cannot be mentally weak (Coach6).

Athletes are afraid of being perceived as weak (Coach 7).

Because a scientific understanding of mental health is not fully accepted by both athletes and coaches, most of them believe that players don't have mental health problems. When experiencing mental health issues, they avoid admitting and facing them (Team official 4).

Elite athletes prefer not to admit that they have mental health issues. They prefer to recognise their difficulties in terms of skills or physical aptitudes than psychological problems. They may feel embarrassed or ashamed (Team official 3).

#### Positive attitudes

Some of the athletes and coaches suggested that there should be a positive attitude towards those who seek help for mental health issues. The athletes felt that they faced more severe pressure than non-athletes, and the coaches noted that every athlete experienced emotional adversity during training and competition.

I think it is normal for athletes to experience mental health difficulties in training and competition. We suffer from the pressure of competition. We're just normal people (Athlete19).

From my perspective, I think this is a normal thing. Because everyone encounters adversities in their growth phase, both physically and mentally. I think we should face difficulties and find effective ways to solve problems (Coach 8).

### Help-seeking intentions

The interviewees in all three groups believed that some elite athletes have the intention of seeking help when experiencing mental health issues. However, some of the coaches and team officials indicated that they preferred their athletes not to seek such help from others. In some cases, their reasons were related to stigmas; in other cases, they simply did not know where the athletes could go to seek help.

#### Seeking help from others

The athletes and coaches agreed that many athletes intend to seek help when experiencing mental health issues. Furthermore, the team officials indicated that elite athletes tend to seek help through informal approaches (such as talking to teammates or parents), rather than using formal approaches (i.e., coaches). The participants also reported that athletes tend to avoid seeking help from sports psychologists, perhaps due to concerns over public or self-stigmas.

I prefer to seek help from my teammates who could understand this feeling and share my feelings. They can help me to analyze and solve the problem through their experience or understanding (Athlete 12).

One player once said that she intended to seek help from a sport psychologist, to talk about some difficulties related to pre-competitive anxiety. She trained on the national team and felt the pressure from her coach and competitors on the same team (Coach 6).

Some of them (athletes) may ask their coaches for help when struggling with mental health issues, but it mainly depends on the relationship of trust between coaches and athletes (Team official 1).

#### Self-help intention

The interviewees indicated that some elite athletes usually prefer to seek help from “no-one” when experiencing mental health issues. The coaches revealed that athletes, especially males, tend to choose self-help. A possible reason relates to the fear of stigmatisation reported by both coaches and team officials. Another reason mentioned by the team officials was that athletes are not sure where they should go to seek help.

I prefer to cry, sing, or drink. I might not be able to solve my issues effectively, but I prefer to stay alone (Athlete 14).

For male athletes, they prefer self-adjustment because it's difficult to tell others. They believe that they have the ability to solve their problems (Coach 8).

Two possible reasons should be paid into attention. Firstly, they did not know where to find support if they suffering mental health-related issues. Secondly, they might prefer to choose self-help because of embarrassment (Team official 2).

### Help-seeking behaviours

The interviewees in all three groups agreed that some elite athletes suffering from mental health difficulties generally prefer to seek help from informal approaches such as parents, friends, or teammates, before turning to formal approaches such as coaches or sports psychologists. Furthermore, they agreed that self-help is another strategy adopted by some elite athletes.

#### Seeking help from others

The interviewees in all three groups reported that when elite athletes experience mental health issues, they generally seek support from someone whom they trust and regard as supportive. Their usual priority in seeking help is to use informal sources (i.e., teammates, friends, or parents), followed by formal sources (i.e., coaches or sports psychologists). Some of the team officials believed that for issues related to sports performance, athletes should consult sports psychologists only after getting a referral from their coaches.

My teammates supported me. They organised some activities to comfort me, to make me feel better. I also asked my close friends for help with my emotional problems. For example, there was a time when I cried in the training centre because of my performance and the pressure, I felt awful. Then they said “You have no choice; you have to accept it. You are an athlete; you must understand it” (Athlete 14).

For adult athletes, they usually ask me for help. Because they trust me. It may be due to my years of experience. Then we can communicate more freely. If I cannot solve their problems, I will do my best to seek help from others, such as sport psychologists or leaders. For younger players, they usually talk to their parents about mental health concerns. Some parents may mention trust and supporting behaviour, including giving feedback to coaches (Coach12).

There is increasing awareness of mental health issues, which could influence the training offered by some younger coaches. So they suggest their athletes to consult sports psychologists regularly (Team official 1).

#### Self-help strategies

The interviewees from the three groups agreed that elite athletes, especially males, may find it difficult to seek help from others. Therefore, many athletes prefer to engage in self-help. Elite athletes might seek to “put on a brave face” to conceal their emotional vulnerability and mental health difficulties. Some of the interviewed athletes believed that they could fix their mental health issues by themselves, through measures such as reading books or listening to music. Some athletes believed in negative measures such as smoking and drinking alcohol. However, the coaches and team officials agreed that these negative strategies cannot solve athletes' mental health difficulties.

I prefer reading books and jogging to solve my mental health problems. My teammates like to write a diary or listen to songs. Some of my teammates said that drinking alcohol would be an appropriate approach to alleviate their emotional problems, but I think it would just hide their problems rather than solve them (Athlete 10).

They prefer smoking, drinking, and having fun with their friends, as a way of trying to cover it up. But I don't think the issues can be solved effectively that way. It just helps them to alleviate their negative emotions at the times they happen (Coach 5).

I know some players would like to drink to solve their problems. I am not sure whether it is effective or not (Team official 5).

### Chinese sports environment

The participants identified sports environment that can influence athletes' mental health and their capacity to seek help when needed. In general, they believed that supportive relationships and a positive environment from the sports system facilitate help-seeking. In contrast, poor support from significant others, an unhealthy and untrustworthy sports system can negatively impact athletes' mental health and capacity for help-seeking.

#### Social support

Generally, the three groups of participants indicated that encouragement and support from trusted significant others, including parents, teammates, friends, coaches, officials, and sports psychologists, facilitate mental health and help-seeking by athletes.

The coach's attitude was understandable and they were willing to support you (Athlete 2).

Mutual help and encouraging words should be offered by teammates, which facilitate help-seeking. Team awareness and team importance are emphasised in our team (Coach 11).

Mental health and personal development were also important. I would like to give fully supportive sources to assist athletes if they encountering what kinds of adversities or mental health issues (Team official 4).

#### Chinese sports system

The participants expressed some disagreement about whether the Chinese sports system can provide a positive and friendly environment for athletes who have mental health concerns. The interviewees from all three groups generally believed that the Chinese sports system provides a good environment for players. For example, this system offers excellent examples from other successful athletes, good and trusting relationships between athletes and their coaches, supportive training environments, excellent teams and consistent training to build confidence and stress tolerance. However, some of the respondents also indicated concerns over unfriendly and competitive relationships among athletes, a poor coach–athlete relationship, negative and unhealthy environments in sports teams and performance-driven stress in the system. They felt that these issues result in mental health issues and hinder athletes from seeking help.

In our team, one athlete has always been a model, a great example. We can all learn from him. This encourages us to be mentally strong (Athlete 20).

Good relationships among players enabled them to develop mental health. They could support and encourage each other when suffering mental difficulties, and then they might find ways to solve these problems together. While, the bad results would be acquired if the relationships were unfriendly, accordingly (Coach 7).

Some high-level elite athletes with excellent performance played core roles in the team. Others would like to behave in line with the excel model. Additionally, others preferred to seek help from the model when they struggling with difficulties, such as injuries or deficits in mental health-related areas. It benefits for both athletes and whole teams in terms of personal development and performance. Otherwise, the situation might be worse due to the unfriendly relationship or bad model behaviours (Team official 4).

The coach could encourage you if you told him the mental health issues. But it requires good relationships between coaches and athletes. Then, the athletes could talk about the issues openly (Athlete2).

Some athletes who went to the national team did not get along well with the coach, and the quality of their training was weakened. During the training, they might have felt as if they were less valued, and then they probably lost interest in participating (Coach 12).

The positive sports environment means that principles and rules are followed, and rewards and punishments are fairly received. This is conducive to fostering the ability to deal with pressure and cultivate a supportive training environment (Coach 7).

National teams emphasized the importance of mental health in recent years. However, the significance was not admitted by provincial teams. Thus, some of them concealed their personal feelings by only focusing on performance rather than mental health. It was hard for them to talk about mental issues freely and seek help directly (Team official 5).

### Chinese cultural background

Most of the participants, including some of the athletes and coaches and all of the team officials, suggested that the traditional Chinese culture emphasises morality and social responsibility, along with harmony between individuals and their society. These participants felt that this cultural background can improve athletes' mental health. However, some of the athletes and coaches worried that the culture's emphasis on responsibility and “face” can lead athletes to experience a high level of stress related to performance.

Chinese culture can facilitate people's mental health because it teaches us that you must be responsible for your teams and society. Moreover, with the increase of knowledge in the public, [mental health issues] are more accepted by others (Athlete 5).

We suffer from competitive pressure because everyone tells us: “You have the responsibility to fight for our teams”. I cannot tell my feelings to others. You know, in our culture, you are afraid of being perceived as weak (Athlete 10).

What we have learned from the traditional culture, such as responsibilities and the spirit of “never give up”, can foster our ability to overcome difficulties, and contribute to the improvement of mental health and help-seeking behaviours (Coach 6).

During the past decades, mental health was not paid much attention to individuals in our country, not only for coaches and athletes but also for the public. Furthermore, most athletes were unwilling to discuss the issues they experienced, especially related to mental health issues (Coach 2).

Morality, harmony and social responsibility are emphasised by Chinese traditional culture. This philosophy of life, including standards for how to cope with others, how to deal with difficulties, and how to offer information and services to help others, can promote athletes' mental health and encourage them to seek assistance (Team official 4).

## Discussion

The current study aimed to gain a bottom-up understanding of the mental health issues in relation to these athletes' experiences, knowledge, help-seeking attitudes, intentions and behaviours. Seven main themes emerged from the data analysis, namely mental health experiences; MHL; help-seeking attitudes; help-seeking intentions; help-seeking behaviours; Chinese sports environment and Chinese cultural background influencing athletes' mental health and help-seeking. This study provides valuable information about how to promote athletes to seek help when they experiencing mental health issues. Findings of the current study can be used to develop mental health promotion programmes tailored for the needs of Chinese elite athletes.

Regarding experience of mental health and the impact on performance, the participants offered insights into athletes' experiences of mental health issues, which closely associated with performance. The participants reported many of the same experiences of mental health issues that have been indicated by previous studies (1, 20). It should be emphasised that the sleep-related concerns and weight control issues were each noted by only two of the three groups' interviewees. Previous findings have shown that sleep issues can weaken athletic performance in many sports (62, 63), especially if sleep is lost on the nights before competitions (64). Weight control issues was also found to be an important factor that can affect mental health, especially for athletes in weigh-in sports. Although the issues of eating disorders and disordered eating caused by weight control were not mentioned, weight control problems may be the strongest predictors of eating disorders in athletes (65, 66). Research indicated that elite athletes struggled with different mental health issues, which further impacted performance (7). Therefore, the participants indicated that experience of mental health issues, and existing risk factors should be more highly valued in the future studies.

Although MHL was the first step for athletes to seek assistance (36), the current study revealed that athletes had low levels of MHL. This is consistent with previous findings (24, 45, 67). Furthermore, previous studies demonstrated that MHL had a positive relationship with help-seeking (68–70). Since the lower level of MHL and the unlikelihood of help-seeking in elite athletes, MHL is important for this population. This further demonstrated how urgently the Chinese elite athlete population needs pertinent MHL interventions (71, 72). Research revealed that athletes might not know how to recognise mental health issues, mental health risk factors, how to seek mental health information or how to find what help is available (21). Considering the importance of MHL, research has emphasised that it should be enhanced not only in the athletes, but also in the coaches, parents, and working staff (66–69). It would be beneficial for creating a supportive environment for athletes (32). Therefore, the importance of knowledge mental health issues, and awareness of their potential consequences, should be noticed by the sport psychologists and coaches as previous research suggested (42, 73).

Help-seeking attitudes are mainly related to both public and self-stigmas, which further supported the findings of previous studies (26, 33). Due to the public-stigma, athletes who seek help for mental health concerns may be viewed as weak by others (26). Additionally, self-reported difficulties by athletes might be caused by self-stigmas, which caused them shame, embarrassment, or discomfort about seeking help for their issues. Higher levels of public- and self-stigma may inhibit decision-making and service selection processes (74). Therefore, stigmas were emphasised as serious issues that tend to hinder help-seeking by athletes (32, 71). Despite these issues, both athletes and coaches seemed to practise the promotion of positive attitudes towards dealing with mental health issues, which might be due to increasing awareness on the part of the IOC (1). This, of course, enabled growing acceptance for athletes who seek help with mental health issues such as depressive symptoms or anxiety. Although some positive attitudes towards help-seeking were indicated by athletes and coaches in the current study, the effects of public and self-stigmas should be further investigated in future studies.

Help-seeking intentions and behaviours involved either seeking help from others or self-help. Athletes preferred to seek assistance from informal sources (i.e., teammates or friends) rather than formal sources (i.e., coaches or sports psychologists), which is consistent with previous research on help-seeking behaviours (36). As discussed above, stigmas were found to be the main barriers to seeking help. Therefore, self-help intentions and behaviours should be promoted, especially for males (27). Additionally, athletes used both positive and negative self-help strategies, which further supported previous findings (75). Yet, the negative strategies might be ineffective in dealing with mental health issues. Previous studies have demonstrated that negative strategies may simply cover up issues rather than fix them (75). Therefore, future studies should further investigate how to build positive strategies and avoid negative strategies in coping with mental health issues.

Sport environment influence athletes' mental health and help-seeking propensity, which further supported previous research (32, 76, 77). First, considering social support, strong existing support from teammates, parents, coaches, sports psychologists, and team officials would enable them to seek assistance when struggling with mental health. Particularly, social support plays a significant role in helping athletes to cope, reducing the negative effects of stressors and improving mental health (32, 78, 79). A comprehensive framework for providing both formal and informal support could facilitate help-seeking among athletes who encounter mental health issues (21). Regarding the Chinese sports environment, some of the culture's vital components, such as its emphasis on building positive relationships with others (e.g., teammates and coaches) and promoting a positive and harmonious training environment, should be further investigated in future studies (80). Second, considering elite sports environments, some of the culture's vital components, such as its emphasis on building positive relationships with others (e.g., teammates and coaches) and promoting a positive and harmonious training environment, should be further investigated in future studies (80). Furthermore, how to build the links between MHL and mental health should be also noted, which might further contribute to relevant intervention conducted in elite athletes (73).

Culture might be one important factor that cultivates one's resilience to deal with their difficulties (42). Therefore, the importance of both fostering harmony among individuals and within society and promoting morality and social responsibility was highlighted in the participants' discussions (40). However, traditional beliefs and values of Chinese culture may lead to high levels of stigmas for those experiencing mental health difficulties (77). For instance, due to their traditional culture, Chinese people usually support independence or seek mental health treatment through traditional Chinese medicine, believing that it is a form of retribution for their ancestors' misconduct (76, 77). This may cause mental health issues to worsen and recur, in addition to lowering an individual's level of MHL (76, 77). Clearly, stigmas related to mental health should be reduced to effectively improve mental health in China's athlete population (32, 81). Furthermore, a lack of sources to seek help should be highlighted. Previous studies revealed that in the low and middle-income countries, some difficulties such as lack of approaches to acquire sources, inadequate training and knowledge were commonly seen (42). Therefore, future studies aiming at preventing and/or treating mental health issues should be focused both individually and systemically (43). Some collaboration, including sport psychology, sports psychiatry, and clinical psychology, should be encouraged to propose strategies for elite athletes' mental health (43). It is should also be highlighted that the relevant intervention material needed to be translated and adapted due to the culture adaption (42).

### Strengths and limitations

This study has several strengths which should be emphasised. First, the data were collected from athletes, coaches, and team officials, and were analysed by two independent coders. Second, to the best of our knowledge, this is the first study to focus on the mental health issues and help-seeking tendencies of Chinese elite athletes while considering Chinese culture and the unique Whole Nation sport system. Third, team officials were involved in this study, as these officials play significant roles in promoting policies and strategies in Chinese systems of training in sports and culture. Therefore, the viewpoints of team officials provided a unique and important perspective on mental health and help-seeking in elite sports.

This study also has limitations. First, the findings could have been affected by male gender bias, due to the male-dominated sport culture; all of the participating team officials were males, and eight of the coaches were males. Although this sample was broadly representative of the gender pattern among team officials and coaches in the sports concerned, it would also be beneficial to solicit the opinions of more female coaches and team officials, as gender differences may affect the findings. Second, the participants came from two Chinese provinces only, and did not reflect the geographic diversity of the sports population, thereby potentially limiting the study's external validity. Third, the data were collected before the COVID-19 epidemic, so we did not consider the impact of it. Last, the experiences of mental health issues were not diagnosed by the existing instruments, such as the DSM-5 from American Psychiatric Association (82), or the ICD-11 from WHO (83). Therefore, it's hard to identify the mental health condition as suggested by the International Society of Sport Psychology, such as clinical mental health disorders, subclinical mental ill health, the human condition, and the athlete condition (2, 43).

## Conclusion

The current study revealed that Chinese elite athletes suffered from various mental health issues and athletes' MHL levels, help-seeking attitudes and intentions, Chinese sports environments, and Chinese cultural background could impact their help-seeking behaviours. Support for Chinese elite athletes' mental health and help-seeking requires improvement.

## Data availability statement

The raw data supporting the conclusions of this article will be made available, upon request to the corresponding author.

## Ethics statement

Ethical approval was received for this study from the Research Ethics Committee of Hong Kong Baptist University. Written informed consent for participation in this study was provided by the participants (aged over 18) or participants' legal guardians /next of kin (aged under 18).

## Author contributions

DB: Writing – original draft, Writing – review & editing. C-QZ: Methodology, Writing – review & editing. J-DL: Methodology, Writing – review & editing. ZHa: Methodology, Resources, Writing – review & editing. XW: Methodology, Writing – review & editing. ZHu: Funding acquisition, Methodology, Supervision, Writing – review & editing.

## References

[1] Reardon CL Hainline B Aron CM Baron D Baum AL Bindra A. Mental health in elite athletes: International Olympic Committee consensus statement (2019). Br J Sports Med. (2019) 53:667–99. 10.1136/bjsports-2019-100715 31097450

[2] Henriksen K Schinke R Moesch K McCann S Parham WD Larsen CH. Consensus statement on improving the mental health of high performance athletes. Int J Sport Exerc Psychol. (2019) 18:553–60. 10.1080/1612197X.2019.1570473

[3] Moesch K Kenttä G Kleinert J Quignon-Fleuret C Cecil S Bertollo M. FEPSAC position statement: Mental health disorders in elite athletes and models of service provision. Psychol Sport Exerc. (2018) 38:61–71. 10.1016/j.psychsport.2018.05.013

[4] Chang C Putukian M Aerni G Diamond A Hong G Ingram Y. Mental health issues and psychological factors in athletes: Detection, management, effect on performance and prevention: American Medical Society for Sports Medicine Position Statement-Executive Summary. Br J Sports Med. (2020) 54:216–20. 10.1136/bjsports-2019-101583 31810972

[5] Si G Li X Huang Z Wang D Wang Y Liu JD. The mental health of Chinese elite athletes: revisiting the assessment methods and introducing a management framework. Int J Sport Exerc Psychol. (2021) 1–15. 10.1080/1612197X.2021.1907769

[6] Van Slingerland KJ Durand-Bush N Bradley L Goldfield G Archambault R Smith D. Canadian Centre for Mental Health and Sport (CCMHS) Position Statement: principles of mental health in competitive and high-performance sport. Clin J Sport Med. (2019) 29:173–80. 10.1097/JSM.0000000000000665 31033609

[7] Schinke RJ Stambulova NB Si G Moore Z. International society of sport psychology position stand: Athletes' mental health, performance, and development. Int J Sport Exerc Psychol. (2018) 16:626–9. 10.1080/1612197X.2017.1295557

[8] Gouttebarge V Bindra A Blauwet C Campriani N Currie A Engebretsen L. International Olympic Committee (IOC) Sport Mental Health Assessment Tool 1 (SMHAT-1) and Sport Mental Health Recognition Tool 1 (SMHRT-1): Towards better support of athletes' mental health. Br J Sports Med. (2021) 55:30–7. 10.1136/bjsports-2020-102411 32948518 PMC7788187

[9] Kumar S Devi G. Sports Performance and Mental health of Athletes. Sports Sci Health Adv. (2023) 1:46–9. 10.60081/SSHA.1.1.2023.46-49

[10] Daley MM Shoop J Christino MA. Mental health in the specialized athlete. Curr Rev Musculoskelet Med. (2023) 16:410–8. 10.1007/s12178-023-09851-1 37326758 PMC10427563

[11] Mountjoy M Junge A Bindra A Blauwet C Budgett R Currie A. Surveillance of athlete mental health symptoms and disorders: a supplement to the International Olympic Committee's consensus statement on injury and illness surveillance. Br J Sports Med. (2023) 57:1351–60. 10.1136/bjsports-2022-106687 37468210

[12] Kussman A Choo HJ. Mental health and disordered eating in athletes. Clin Sports Med. (2024) 43:71–91. 10.1016/j.csm.2023.07.001 37949515

[13] Schaal K Tafflet M Nassif H Thibault V Pichard C Alcotte M. Psychological balance in high level athletes: Gender-based differences and sport-specific patterns. PLoS ONE. (2011) 6:e0019007. 10.1371/journal.pone.0019007 21573222 PMC3087722

[14] Du Preez EJ Graham KS Gan TY Moses B Ball C Kuah DE. Depression, anxiety, and alcohol use in elite rugby league players over a competitive season. Clin J Sport Med. (2017) 27:530–5. 10.1097/JSM.0000000000000411 28107218

[15] Hammond T Gialloreto C Kubas H Davis H. The prevalence of failure based depression among elite athletes. Clin J Sport Med. (2013) 23:273–7. 10.1097/JSM.0b013e318287b870 23528842

[16] Bratland-Sanda S Sundgot-Borgen J. Eating disorders in athletes: overview of prevalence, risk factors and recommendations for prevention and treatment. Eur J Sport Sci. (2013) 13:499–508. 10.1080/17461391.2012.740504 24050467

[17] NCAA. NCAA GOALS. Study 2015. Indianapolis, IN (2016).

[18] Liu Z. An Investigation and Analysis of Characteristics About Exercise-Induced Insomnia. Suzhou: Suzhou University (2012).

[19] Pluhar E McCracken C Griffith KL Christino MA Sugimoto D Meehan WP. Team sport athletes may be less likely to suffer anxiety or depression than individual sport athletes. J Sports Sci Med. (2019) 18:490–6. 31427871 PMC6683619

[20] Rice SM Purcell R De Silva S Mawren D McGorry PD Parker AG. The mental health of elite athletes: a narrative systematic review. Sports Med. (2016) 46:1333–53. 10.1007/s40279-016-0492-2 26896951 PMC4996886

[21] Gulliver A Griffiths KM Christensen H. Barriers and facilitators to mental health help-seeking for young elite athletes: a qualitative study. BMC Psychiatry. (2012) 12:157. 10.1186/1471-244X-12-157 23009161 PMC3514142

[22] Cosh SM McNeil DG Jeffreys A Clark L Tully PJ. Athlete mental health help-seeking: A Systematic review and meta-analysis of rates, barriers and facilitators. Psychol Sport Exerc. (2024) 71:102586. 10.1016/j.psychsport.2023.102586 38128709

[23] Rickwood D Thomas K. Conceptual measurement framework for help-seeking for mental health problems. Psychol Res Behav Manag. (2012) 5:173–83. 10.2147/PRBM.S38707 23248576 PMC3520462

[24] Guo Y. Anaysis of Relevant Factors to Excellent Sportsmen's Mental Health. Zhengzhou: Zhengzhou University (2008).

[25] Breslin G Haughey T O'Brien W Robertson A Caulfield L Lawlor M. Increasing athlete knowledge of mental health and intentions to seek help: the state of Mind Ireland (SOMI) pilot program. J Clin Sport Psychol. (2018) 12:39–56. 10.1123/jcsp.2016-0039

[26] Gulliver A Griffiths KM Christensen H. Perceived barriers and facilitators to mental health help-seeking in young people: a systematic review. BMC Psychiatry. (2010) 10:113. 10.1186/1471-244X-10-113 21192795 PMC3022639

[27] Wood S Harrison LK Kucharska J. Male professional footballers' experiences of mental health difficulties and help-seeking. Phys Sportsmed. (2017) 45:120–8. 10.1080/00913847.2017.1283209 28095726

[28] Jorm AF Korten AE Jacomb PA Christensen H Rodgers B Pollitt P. “Mental health literacy”: A survey of the public's ability to recognise mental disorders and their beliefs about the effectiveness of treatment. Med J Aust. (1997) 166:182–6. 10.5694/j.1326-5377.1997.tb140071.x 9066546

[29] Wahto RS Swift JK Whipple JL. The role of stigma and referral source in predicting college student-athletes' attitudes toward psychological help-seeking. J Clin Sport Psychol. (2016) 10:85–98. 10.1123/JCSP.2015-0025

[30] Watson JC. College student-athletes' attitudes toward help-seeking behavior and expectations of counseling services. J Coll Stud Dev. (2005) 46:442–9. 10.1353/csd.2005.004432877631

[31] Corrigan P. How stigma interferes with mental health care. Am Psychol. (2004) 59:614–25. 10.1037/0003-066X.59.7.614 15491256

[32] Bu D Han Z Zhang C Liu J Huang Z Wang A. The effect of a mental health literacy intervention on Chinese team officials and staff in elite sports : a two-arm non-randomised controlled trial. Int J Sport Exerc Psychol. (2023) 1–18. 10.1080/1612197X.2023.2269156

[33] Bauman NJ. The stigma of mental health in athletes: are mental toughness and mental health seen as contradictory in elite sport? Br J Sports Med. (2016) 50:135–6. 10.1136/bjsports-2015-095570 26626270

[34] Breslin G Haughey TJ Donnelly P Kearney C Prentice G. Promoting mental health awareness in sport clubs. J Public Ment Health. (2017) 16:55–62. 10.1108/JPMH-08-2016-0040

[35] Breslin G Kearney C Haughey T. Mental health and well-being in sport: a pilot educational programme for clubs. Ulster University (2015).27409075

[36] Rickwood D Deane FP Wilson CJ Ciarrochi J. Young people's help-seeking for mental health problems. Aust E J Adv Ment Health. (2005) 4:218–51. 10.5172/jamh.4.3.218

[37] Ajzen I. The theory of planned behavior. Organ Behav Hum Decis Process. (1991) 50:179–211. 10.1016/0749-5978(91)90020-T

[38] Davies EB. Development of an Online Intervention to Increase Mental Health Literacy and Promote Self-Management of Depression in University Students. Nottingham: University of Nottingham (2015).

[39] American Psychological Association. Division of Counseling Psychology, Committee on Definition. Counseling psychology as a specialty. Am Psychol. (1956) 11:282–5. 10.1037/h0044771

[40] Duan C. Culture and mental health counseling: A reflective view based on observations in China. Athens J Soc Sci. (2018) 6:1–17. 10.30958/ajss.6-1-5

[41] Committee of National Health and Family Planning. Guidance on Strengthening Mental Health Services. National Disease Control and Prevention 2016 (No.77) (2016). Available online at: https://bit.ly/2Ga9Ai8 (accessed April 20, 2018).

[42] Rathod S Persaud A Naeem F Pinninti N Tribe R Eylem O. Culturally adapted interventions in mental health: global position statement. World Cult Psychiatry Res Rev. (2019) 14:21–9. 10.2147/NDT.S138430 29379289 PMC5757988

[43] Gorczynski P Currie A Gibson K Gouttebarge V Hainline B Castaldelli-Maia JM. Developing mental health literacy and cultural competence in elite sport. J Appl Sport Psychol. (2020) 33:387–401. 10.1080/10413200.2020.1720045

[44] Coyle M Gorczynski P Gibson K. “You have to be mental to jump off a board any way”: elite divers' conceptualizations and perceptions of mental health. Psychol Sport Exerc. (2017) 29:10–8. 10.1016/j.psychsport.2016.11.005

[45] Ferguson HL Swann C Liddle SK Vella SA. Investigating youth sports coaches perceptions of their role in adolescent mental health. J Appl Sport Psychol. (2018) 31:235–52. 10.1080/10413200.2018.1466839

[46] Willig C. Introducing Qualitative Research in Psychology. Maidenhead: Open University Press (2013).

[47] Bhaskar R. Reclaiming Reality: A Critical Introduction to Contemporary Philosophy. London: Verso (1989).

[48] Poucher ZA Tamminen KA Caron JG Sweet SN. Thinking through and designing qualitative research studies: a focused mapping review of 30 years of qualitative research in sport psychology. Int Rev Sport Exerc Psychol. (2019) 13:163–86. 10.1080/1750984X.2019.1656276

[49] Shu Y. Investigation and Intervention of Mental Health on Elite Athlete: In View of Dual-Factor Model of Mental Health. Shanghai: Shanghai Sport University (2015).

[50] Mason M. Sample size and saturation in PhD studies using qualitative interviews. Forum Qual Soc Res. (2010) 11:8. 10.17169/fqs-11.3.1428

[51] Elo S Kyngäs H. The qualitative content analysis process. J Adv Nurs. (2008) 62:107–15. 10.1111/j.1365-2648.2007.04569.x 18352969

[52] Hsieh HF Shannon SE. Three approaches to qualitative content analysis. Qual Health Res. (2005) 15:1277–88. 10.1177/1049732305276687 16204405

[53] Budd RW Thorp RK Donohew L. Content Analysis of Communications. New York, NY: Macmillan (1967).

[54] Lindkvist K. Approaches to textual analysis. In:Rosengren KE, editor. Advances in Content Analysis. Beverly Hills, CA: Sage (1981).

[55] McTavish DG Pirro EB. Contextual content analysis. Qual Quant. (1990) 24:245–65. 10.1007/BF00139259

[56] Tesch R. Qualitative Research: Analysis Types and Software Tools. Bristol, PA: Falmer (1990).

[57] Kondracki NL Wellman NS Amundson DR. Content analysis: review of methods and their applications in nutrition education. J Nutr Educ Behav. (2002) 34:224–30. 10.1016/S1499-4046(06)60097-3 12217266

[58] Côté J Salmela JH Baria A Russell SJ. Organizing and interpreting unstructured qualitative data. Sport Psychol. (1993) 7:127–37. 10.1123/tsp.7.2.127

[59] Côté J Salmela JH. A decision-making heuristic for the analysis of unstructured qualitative data. Percept Mot Skills. (1994) 78:465–6. 10.2466/pms.1994.78.2.465

[60] Yardley L. Dilemmas in qualitative health research. Psychol Health. (2000) 15:215–28. 10.1080/08870440008400302

[61] Smith JA. Qualitative Psychology: A Practical Guide to Research Methods. Sage (2015).

[62] Calogiuri G Weydahl A. Effects of sleep loss on the rest-activity circadian rhythm of helpers participating in continuous dogsled races. Biol Res Nurs. (2014) 16:123–33. 10.1177/1099800412475077 23389400

[63] Dunican IC Martin DT Halson SL Reale RJ Dawson BT Caldwell JA. The effects of the removal of electronic devices for 48 hours on sleep in elite judo athletes. J Strength Cond Res. (2017) 31:2832–9. 10.1519/JSC.0000000000001697 28081034

[64] Roberts SSH Teo WP Warmington SA. Effects of training and competition on the sleep of elite athletes: a systematic review and meta-analysis. Br J Sports Med. (2019) 53:513–22. 10.1136/bjsports-2018-099322 30217831

[65] Anderson RJ Pierce D. Assumptions associated with mental health literacy training-insights from initiatives in rural Australia. Adv Ment Health. (2012) 10:258–67. 10.5172/jamh.2012.10.3.258

[66] Rousselet M Guérineau B Paruit MC Guinot M Lise S Destrube B. Disordered eating in French high-level athletes: Association with type of sport, doping behavior, and psychological features. Eat Weight Disord. (2017) 22:61–8. 10.1007/s40519-016-0342-0 27838862

[67] Han Z Wang DD Ouyang LQ Niu PC Yun ZM. Adaptation and psychometric properties of Mental Health Literacy Scale in Chinese elite athletes. Hubei Sport Sci. (2019) 38:226–9.

[68] O'Connor M Casey L. The Mental Health Literacy Scale (MHLS): a new scale-based measure of mental health literacy. Psychiatry Res. (2015) 229:511–6. 10.1016/j.psychres.2015.05.064 26228163

[69] Online R Wilson CJ Deane FP Ciarrochi JV Rickwood D. Measuring help seeking intentions: properties of the General Help Seeking Questionnaire. Can J Couns. (2005) 39:15–28.

[70] Smith CL Shochet IM. The impact of mental health literacy on help-seeking intentions: results of a pilot study with first year psychology students. Int J Ment Health Promot. (2011) 13:14–20. 10.1080/14623730.2011.9715652

[71] Bu D. Mental Health Literacy and Help-Seeking in Chinese Elite Athletes. Hong Kong: Hong Kong Baptist University (2021).

[72] Bu D Chung PK Zhang C Liu J Wang X. Mental health literacy intervention on help-seeking in athletes: a systematic review. Int J Environ Res Public Health. (2020) 17:1–18. 10.3390/ijerph17197263 33020448 PMC7579198

[73] Gorczynski P Gibson K Thelwell R Papathomas A Harwood C Kinnafick F. The BASES expert statement on mental health literacy in elite sport. Sport Sci. (2019) 59:6–7.

[74] Cauce AM Domenech-Rodríguez M Paradise M Cochran BN Shea JM Srebnik D. Cultural and contextual influences in mental health help seeking: a focus on ethnic minority youth. J Consult Clin Psychol. (2002) 70:44–55. 10.1037//0022-006X.70.1.44 11860055

[75] Butler-Coyne H Shanmuganathan-Felton V Taylor J. Mental health in equestrian sport. J Clin Sport Psychol. (2018) 13:405–20. 10.1123/jcsp.2018-0002

[76] Wei S Zhuozhuo S Wang S Hall BJ. Barriers to professional mental health help-seeking among Chinese adults: a systematic review. Front Psychiatry. (2020) 11:442. 10.3389/fpsyt.2020.00442 32508688 PMC7251144

[77] Wong DFK Tsui HKP Pearson V Chen EYH Chiu SN. Family burdens, Chinese health beliefs, and the mental health of Chinese caregivers in Hong Kong. Transcult Psychiatry. (2004) 41:497–513. 10.1177/1363461504047932 15709648

[78] Saha R Huebner ES Hills KJ Malone PS Valois RF. Social coping and life satisfaction in adolescents. Soc Indic Res. (2014) 115:241–52. 10.1007/s11205-012-0217-3

[79] Wright KB Rosenberg J Egbert N Ploeger NA Bernard DR King S. Communication competence, social support, and depression among college students: a model of facebook and face-to-face support network influence. J Health Commun. (2013) 18:41–57. 10.1080/10810730.2012.688250 23030518

[80] Si G Jiang X. Exploration on social-cultural meridians of Chinese athletes' psychological training. J Clin Sport Psychol. (2011) 5:325–8. 10.1123/jcsp.5.4.325

[81] Larkin D Levy AR Marchant D Martin C. When winners need help: Mental health in elite sport. Psychologist. (2017) 30:1–8.

[82] American Psychiatric Association. Diagnostic and Statistical Manual of Mental Disorders Fifth Edition. Arlington, VA: American Psychiatric Publishing (2013).

[83] WHO. International Classification of Diseases for Mortality and Morbidity Statistics (11th Revision) (2018). Available online at: https://icd.who.int/browse11/l-m/en

